# Angle-Resolved Cathodoluminescence Interferometry of Plasmonic and Dielectric Scatterers

**DOI:** 10.1021/acs.nanolett.5c02952

**Published:** 2025-09-18

**Authors:** Evelijn Akerboom, Hiroshi Sugimoto, Minoru Fujii, F. Javier García de Abajo, Albert Polman

**Affiliations:** † Center for Nanophotonics, NWO-Institute AMOLF, Science Park 104, 1098 XG Amsterdam, The Netherlands; ‡ Department of Electrical and Electronic Engineering, Graduate School of Engineering, Kobe University, Rokkodai, Nada, Kobe 657-8501, Japan; § 172281 ICFO-Institut de Ciencies Fotoniques, The Barcelona Institute of Science and Technology, 08860 Castelldefels, Barcelona, Spain; ∥ ICREA-Institució Catalana de Recerca i Estudis Avanc̨ats, Passeig Lluís Companys 23, 08010 Barcelona, Spain

**Keywords:** angle-resolved cathodoluminescence, interferometry, metrology, Fourier transform, plasmonics

## Abstract

We demonstrate angle-resolved cathodoluminescence (CL) interferometry from electron-beam-excited plasmonic and dielectric nanostructures placed above a Au-coated substrate. We use 20–30 keV electrons to coherently excite plasmon-mediated radiation, which interferes with its mirror image, providing a method to determine the particle-substrate spacing. In an aloof excitation geometry, transition radiation emitted from the Au substrate adds to the interferogram and provides a means to probe the electron traveling time. The measured CL interferograms are in excellent agreement with a scattering and interferometry model in which a single electron coherently launches plasmons at two separate locations. Polarization-resolved CL measurements confirm the interferometric scattering model. Electron-excited Si Mie scatterers show interferograms modulated with resonantly enhanced emission. CL interferometry enables accurate measurement of critical distances in nanoscale geometries, in particular along the electron-beam direction, which are not easily accessible in electron microscopy, while offering a platform for studying optical interference in complex geometries.

## Graphical abstract

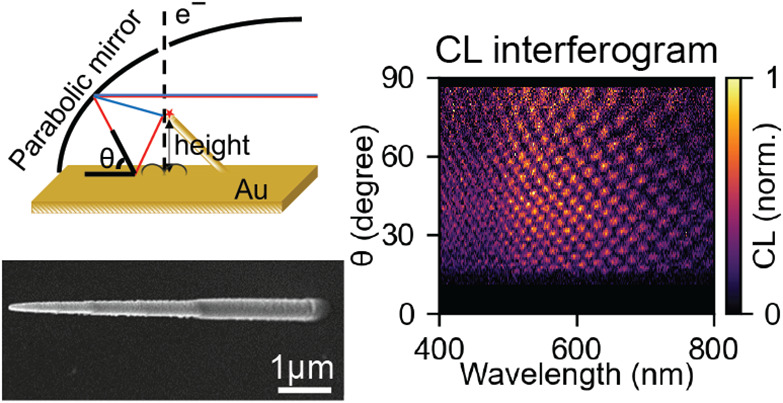

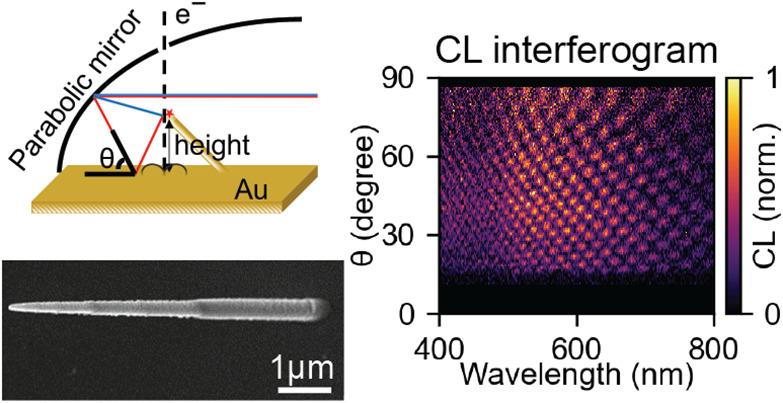

Optical interference is a powerful concept that provides key insights in the properties of light by probing the way differences in phase and amplitude of optical waves vary in space and time. Precise measurements of this phenomenon allow for performing high-precision metrology. With the growing importance of optical metamaterials and nanoscale metastructures, the use of optical metrology is becoming increasingly relevant to probe features at the nanoscale.^1^ For example, the integration of optical metamaterials can help improve the performance of photovoltaics and photocatalysts,^2–4^ provided their geometries are precisely controlled. Likewise, optical metasurfaces can serve as flat optical components, with their precise functionality determined by precision in manufacturing,^5^ while plasmonic antennas that control the directionality of emission in lasers and light-emitting diodes must be accurately shaped and positioned to operate efficiently.^6^ Developing methods to probe the structure and dimensions of nanoscale objects is therefore of great relevance.

In this context, scanning electron microscopy (SEM) is often used to image two- and three-dimensional structured surfaces. SEM collects secondary electrons created by an electron-beam (e-beam) that is raster-scanned over the surface. Conventional SEM typically provides two-dimensional surface information, but several methods have been developed to extract additional insight into the third dimension. One approach is to vary the sample inclination or use beam tilting in TEM tomography, enabling full 3D reconstruction of nanostructures.^7^ This method can yield complete tomographic information but is limited to samples that are thin enough for electron transmission, which often require specialized preparation. Another approach, exemplified by photometric methods, analyzes variations in surface contrast detected at multiple positions relative to the sample. Although this technique can measure surface gradients with high precision, it cannot resolve structures along the electron beam direction, making it unsuitable for stacked layers or situations where the absolute depth along the beam path is required.^8^ Furthermore, deriving precise length information is challenging in SEM, as charging and local beam deflection affect the recorded images. Moreover, calibration of absolute length scales in SEM can have significant errors. Finally, the use of optical methods using, for example, laser excitation to probe nanoscale features in optical metasurfaces is limited by diffraction affecting the light intensity distribution at the surface.

Here, we introduce cathodoluminescence (CL) interferometry as a method to determine the distance between nanophotonic objects and a substrate using e-beam excitation and collection of the angle-resolved emitted CL radiation. Previously, CL interference has been demonstrated in Smith-Purcell radiation^9,10^ or the coherent excitation of surface plasmon polaritons on a Au surface.^11^ Polarized light emission has been achieved using a bullseye structure,^12^ while a spirally structured surface creates optical vortex beams.^13^ Additionally, electron-excited plasmonic Yagi-Uda nanoparticle antenna arrays showed directional emission due to interference of different modes,^14^ and CL holography was demonstrated to measure the phase of plasmon scattering by interference between transition radiation and radiation emitted from a single plasmonic scatterer.^15^ In all these scenarios, a single point of electron impact led to the excitation of multiple radiation sources, which then interfere in the far field. Interference was also observed in experiments on the radiation from electron-excited surface-plasmon polaritons and exciton polaritons in WSe_2_ and h-BN thin layers.^16,17^

In this work, we study the interference of plasmonic scatterers that are separately excited by a single electron at different moments in time. We collect the angle- and wavelength-resolved CL in the far field.^18,19^ Our experiments take advantage of the fact that the e-beam provides a controlled and highly localized source of optical excitation to measure an area of interest. Far-field interference of radiation originating from plasmon excitation provides a powerful means to determine spatial distances at nanoscale precision because the difference between the excitation times translates into a relative phase in the emission amplitudes as a function of emission frequency and angle. Furthermore, CL interferometry can provide information on the coherence of plasmon radiation generated by high-energy electrons.

Figure 1a shows a schematic of our CL interferometry experiments and a SEM image of a free-standing Pt nanopillar fabricated using e-beam-induced deposition (EBID) from a Pt-organometallic precursor. The nanopillar is grown in an SEM at a 45° tilt angle on a Au-coated Si substrate and the entire sample is coated with another 50 nm of sputtered Au (see Supporting Information S8). The e-beam (spot size ∼ 5 nm) is placed on or near the top of the nanopillar. We then perform angle- and wavelength-resolved CL spectroscopy in an SEM equipped with a parabolic mirror placed in between the sample and the electron column. The mirror collects the emitted radiation, and, due to the focusing properties of its parabolic shape, angular information is maintained. Using a vertical slit, we select a narrow range of azimuthal angles (parallel to the length of the pillar) and project these onto a spectrometer to simultaneously retrieve spectrally and angularly resolved data (see Supporting Information S8).

**Figure 1:**
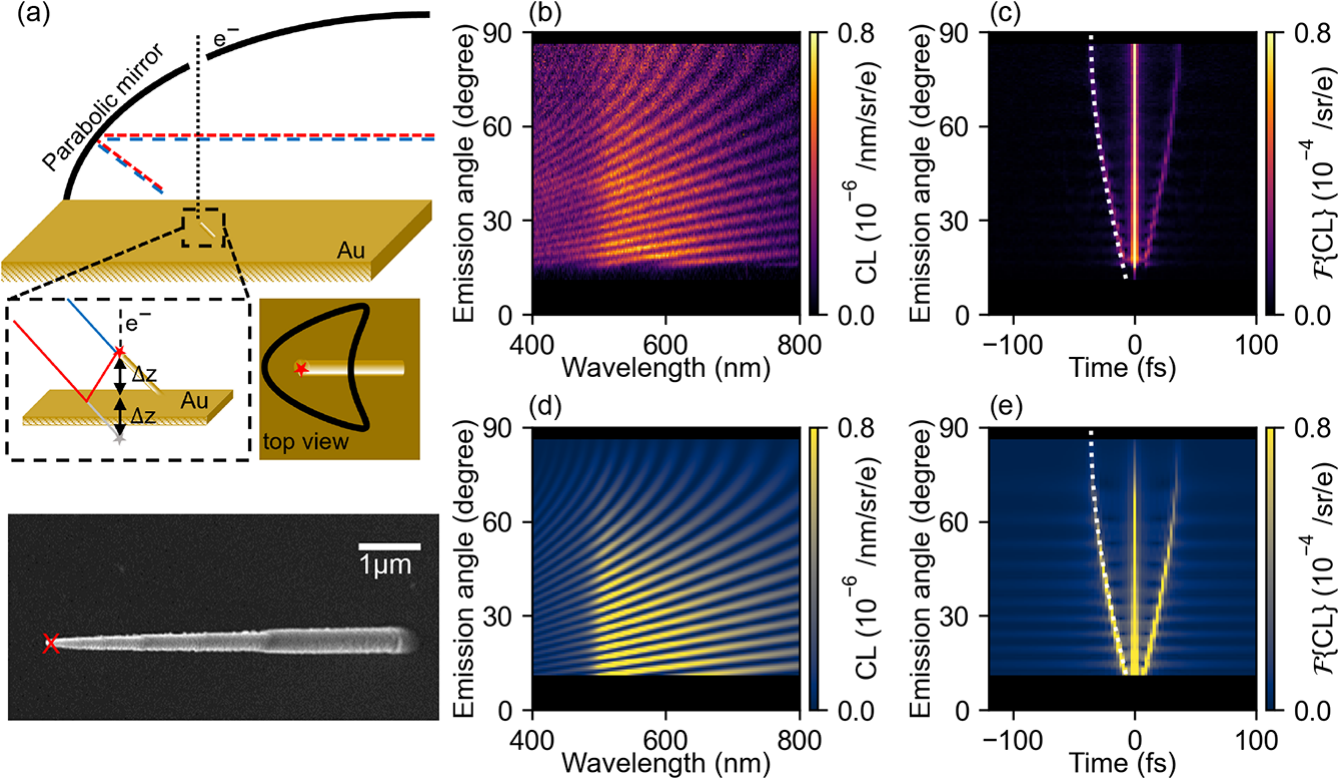
Demonstration of CL interferometry. (a) Schematic representation of the experimental setup and a top-view SEM image of the fabricated sample, with a red cross indicating the impact position of the e-beam. 30 keV electrons excite the tip of a Au pillar, producing light emission, which is collected by the parabolic mirror and directed toward the optical detection system. There are two possible light pathways (directly collected tip emission (blue) and collection of its reflection (red)). The path length difference depends on the emission angle (θ). (b) Experimentally measured angle- and wavelength-resolved CL intensity. (c) Fourier transform of (b) to the time domain. (d) Calculated angle- and wavelength-resolved CL spectra. (e) Fourier transform of (d) to the time domain. The white dashed lines in (c,e) correspond to the time delay given by eq 1 for a pillar with Δ*z* = 5.37 μm.

Figure 1b shows the wavelength and angle-resolved intensity measured over a range from θ = 10° (sideways to the left in Figure 1a) to θ = 90° (corresponding to the surface normal, upward direction). These data are acquired for an e-beam that intersects the pillar at its center, such that it directly excites the tip and does not continue beyond the structure. A clear far-field interference pattern is observed, whose period in the spectral domain strongly depends on the emission angle. We assign these features to the interference of two sources of radiation as represented in Figure 1a: a contribution from the plasmon scatterer directly to the detector (in blue), and another one associated with the plasmon radiation from the same source that is reflected at the metallic surface (in red). The time difference for emission from the two sources is

1 $$\Delta t\left(\theta \right)=\frac{2\Delta z\sin\left(\theta \right)}{c}$$

where Δ*z* is the height of the pillar and *c* the speed of light. For small angles, we observe a long spectral interference period, due to the small path length difference between the two radiation sources. In contrast, at 90° (perpendicular to the surface), the path length difference reaches a maximum (Δ*t* = 2Δ*z/c*) corresponding to the shortest spectral interference period, all in agreement with the interference model. To analyze the data, we Fourier-transform the spectra and move to the time domain for every angle. The result is shown in Figure 1c, where the time delay calculated from eq 1 as a function of angle is overlaid and represents the data very well by fitting the pillar height to 5.37 μm, with a fitting error of less than 1 nm (see Supporting Information S4 for the fitting procedure). We then use this value to compute the angle- and wavelength-dependent CL emission intensity by summing the calculated individual electric-field distributions in the far field (see Supporting Information S1), and the corresponding Fourier transform (see Figures 1d, and e, respectively). We approximate the tip of the nanopillar as a large gold nanoparticle that supports a localized dipolar surface-plasmon resonance around 600 nm (see Supporting Information S2 for CL spectra of a free-standing nanopillar). In the calculations, we use a Mie-based model^20^ to obtain the CL emission from a 150 nm-diameter spherical Au particle excited by an electron at 50 nm from the center, using the dielectric function from ref 21.

Comparing the experimental results to the calculations, several interesting features are observed. First, both theory and experiment show a broad resonance in the spectral intensity around a wavelength of 600 nm with a line width of ∼200 nm, which we attribute to localized dipolar surface plasmons of Au at the tip. Furthermore, in both experiment and calculation, we observe a slight decrease in intensity as the emission angle increases. We assign this to the effect of directional emission from the tip: the electron mostly couples to the *z*-oriented dipole, which dominantly radiates to lower emission angles.

Next, we study the angle- and wavelength-resolved CL emission in a geometry where the e-beam grazes the pillar at a distance of 5 ± 2.5 nm from the apex’s surface, allowing the electron to excite transition radiation (TR) as it reaches the Au substrate, but previously exciting the pillar as well. Figure 2a shows the schematic and SEM image of the geometry with the impact position indicated. The measured angular spectra for this configuration and their Fourier transforms are shown in Figure 2b,c, respectively. Here, a much more complex interference pattern is observed, especially at low emission angles. This is even clearer in the Fourier-transformed image, where two new interference branches show up at longer times, on either side of the ones found above in Figure 1c. The central branches are fitted with a pillar height of 5.34 μm (see Supporting Information S5 for the fitting procedure).

**Figure 2:**
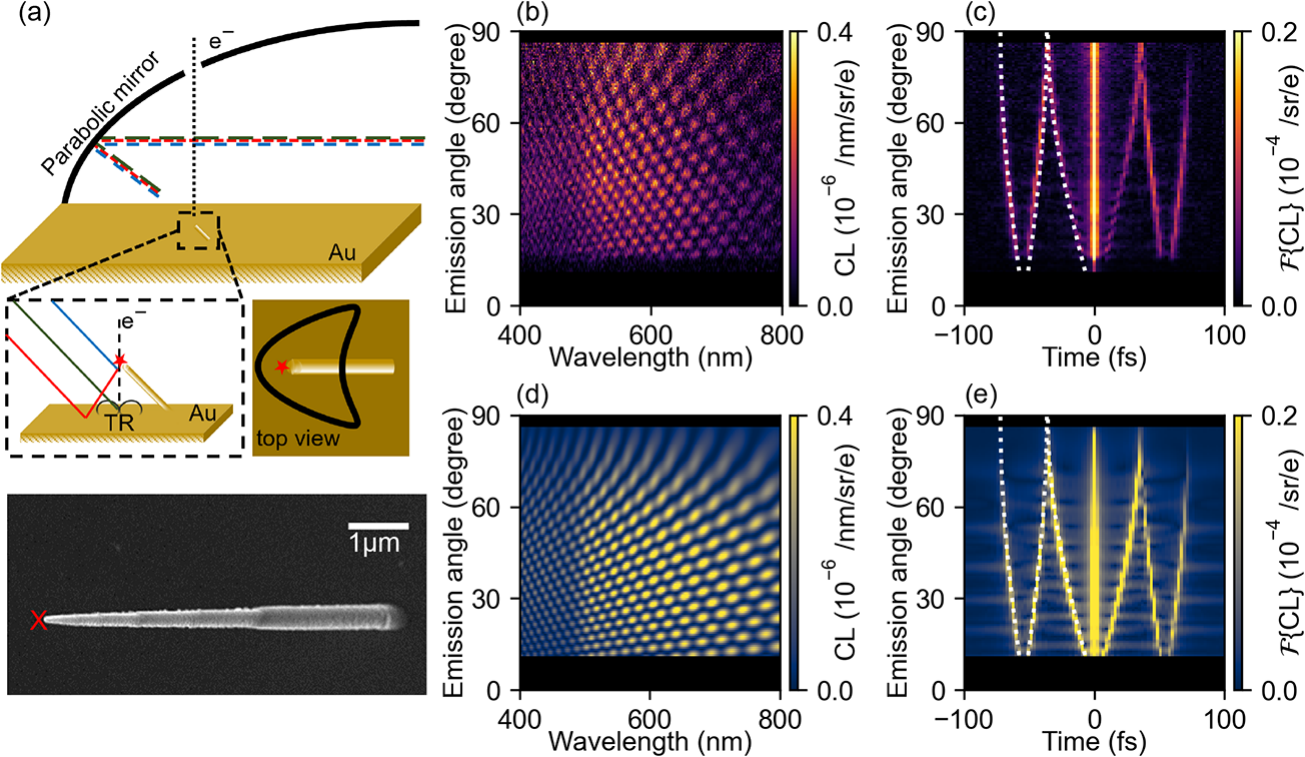
Three-way CL emission interference from tip, image, and TR. (a) Schematic representation of the experimental setup for off-tip excitation and an SEM image of the top view of the fabricated sample, with a red cross indicating the impact parameter of the e-beam. The fast electron (30 keV) grazes the tip of the free-standing Au pillar and later excites TR at the Au substrate. This results in CL emission along three possible pathways: light that travels directly from the tip to the mirror (blue), its reflection from the gold planar surface (red), and the TR emitted from the planar surface (green). (b) Experimentally measured angle- and wavelength-resolved CL intensity. (c) Fourier transform of (b) to the time domain. (d) Calculated angle- and wavelength-resolved CL intensity. (e) Fourier transform of (d). The white dashed lines in the outer branches in (c,e) correspond to the time taken by a 30 keV electron to travel from the tip to the substrate for a pillar with a height of 5.34 μm calculated using eq 2.

We assign the additional interference branches to far-field interference between TR generated by the electron exciting the Au-coated substrate and the two sources that created the interference in Figure 1: direct plasmon radiation from the tip and its reflection from the Au-coated substrate. The expected time delay between radiation from the tip and the TR (Δ*t*_+_), and between reflected radiation from the tip and TR (Δ*t*_–_), is given by

2 $$\Delta t_{\pm }\left(\theta \right)=\frac{\Delta z}{v_{e}}\pm \frac{\Delta z\sin\left(\theta \right)}{c}$$

where the first term in the right-hand side is the time-of-flight of the electron moving with a velocity *v*_*e*_ (≈0.33 *c* at 30 keV). The expected time delay matches very well the outer branches of the Fourier-transformed data (see the white dashed lines in Figure 2c,e). The time delay derived by extrapolating the outer branches to an angle of 0° is 55 fs, which corresponds to an electron velocity that also matches that for 30 keV electrons within 2%. Finally, there is a small part of the TR emission that scatters from the shaft of the nanopillar, although this is not significant in these measurements (see Supporting Information S3).

We extend the model further and calculate the full interferogram by coherently summing the electric fields emitted from the three sources in the far field. For the TR, we use an analytically result^22^ (see Supporting Information S1). In the calculations, we used a pillar height fitted from the inner branches in Figure 2c (Δ*z* = 5.34 μm). The calculated interferogram (Figure 2d) mimics the measured data well, with the strongest signal observed around the plasmon resonance. The angular distribution of the CL emission from the plasmonic tip shows a more Lambertian behavior compared to that in Figure 1. We assign this to a difference in the relative coupling strength of the electron to the in-plane and out-of-plane electric dipoles. From our earlier investigation of the electron-coupling to resonant modes of Au particles, we know that, by exciting close the center of a particle (Figure 1), the out-of-plane dipole is dominant, creating an angular emission profile that is maximum at smaller θ, while for electron grazing next to the particle (Figure 2), both the in-plane and out-of-plane dipole components are excited.^23^ The latter then creates a more Lambertian-like emission profile. Furthermore, in both experiments and calculations in Figure 2, the interference contribution from the TR vanishes at high emission angles, in agreement with the fact that TR has no upward radiation component when the e-beam is normal to the surface. The validity of the multiple-interference model is further supported by the strong similarity between the measured and calculated Fourier transforms shown in Figure 2c and 2e, respectively.

To further evaluate the model, we conduct experiments at an electron energy of 20 keV. The corresponding spectral interferogram and its Fourier transform are shown in Figure 3a and b, respectively. The central branches in the Fourier transform are similar to those for 30 keV electrons, as they correspond to the time delay between the direct and reflected components of the tip radiation, which is independent of the electron velocity. By fitting the Fourier data, we determine the height of this pillar to be 5.81 μm. At the same time, Figure 3b reveals that the outer branches, which correspond to TR components, show up at a larger time delay compared to Figure 2. The extrapolated time delay at an angle of 0° is 71 fs, which corresponds to the electron velocity at 20 keV (*v*_*e*_/*c*≈0.27) within 0.3%.

**Figure 3:**
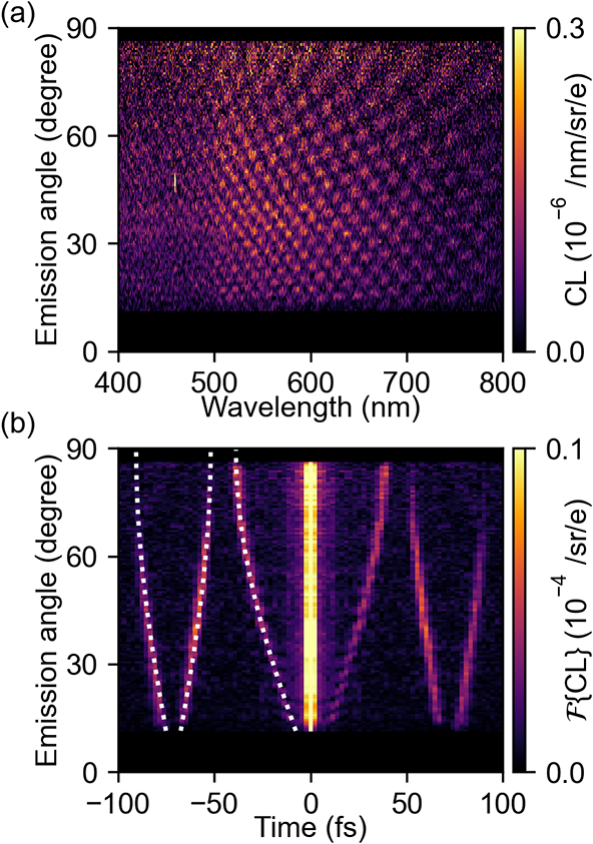
Role of electron velocity. (a) Experimentally measured angle- and wavelength-resolved CL intensity and (b) Fourier transform to the time domain for every emission angle. The white dashed lines in (b) correspond to the time delays for the interfering radiation contributions for a 20 keV electron grazing the pillar at a height of 5.81 μm.

The data in Figures 1–3 clearly show the power of CL interferometry in measuring characteristic length scales in the sample geometry as well as time delays in scattering events due to the electron traveling time. A so far unexplored degree of freedom is the polarization of the emitted light on the interferograms. As the TR component is p-polarized in the scattering plane defined in Figure 1a, it will only interfere with p-polarized emission from the plasmonic tip. To further investigate this effect, we perform polarization-, angle-, and wavelength-resolved CL measurements,^24^ with the e-beam grazing the pillar at a distance of 5 ± 2.5 nm. Figure 4a,b shows the interferograms taken for s- and p-polarized light, respectively. For s-polarized detection, both contributions (pillar interfering with its mirror image and pillar interference with TR) are strongly reduced. This is in accordance with the fact that TR emission is fully p-polarized and the tip emission is mostly p-polarized.

**Figure 4:**
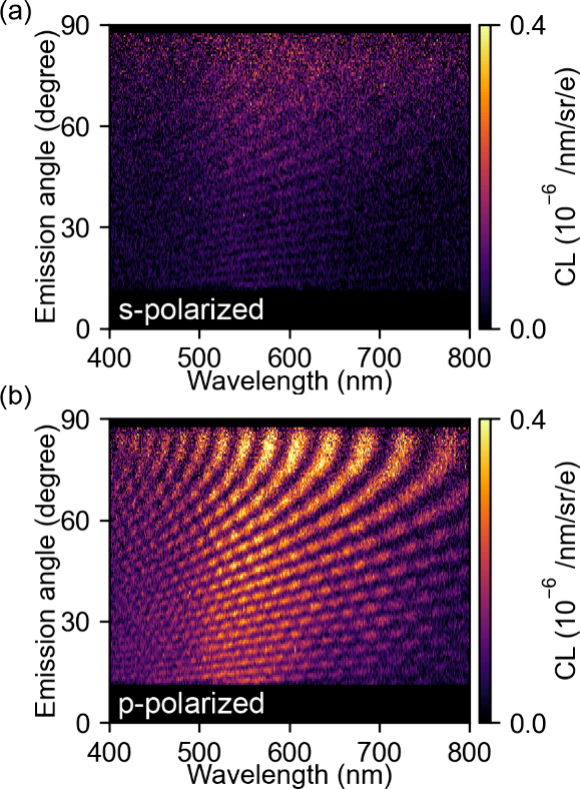
Light polarization analysis. Experimentally measured angle- and wavelength-resolved CL intensity for off-tip excitation for (a) s-polarized and (b) p-polarized light emission using 30 keV electrons.

To further test and exploit the capabilities of the CL interferometry method, we apply it to scattering from dielectric Mie resonators. We use crystalline Si nanospheres^25^ with a diameter of 190 nm, which we place at a controlled distance above a Au-coated Si substrate. Si nanospheres support multiple spectrally narrow Mie resonances with characteristic angular emission patterns. Earlier work has explored the selective coupling between the e-beam and specific Mie modes and how that depends on the impact parameter and electron energy.^26^ Selective excitation of Mie modes allows for tuning the angular CL emission profile by leveraging changes in constructive and destructive interference produced by these modes in the far field.

The sample geometry comprises a 10-μm-diameter W wire coated with the crystalline Si nanospheres. This structure is deposited on a Au-coated Si substrate. A schematic and an SEM image of the W wire with a number of Si nanospheres attached to it is shown in Figure 5a. The distance from a Mie sphere at the edge of the W wire and the substrate is expected to be around 5 μm due to the curvature of the W wire, allowing measurements in a geometry similar to that above for plasmonic scatterers.

**Figure 5:**
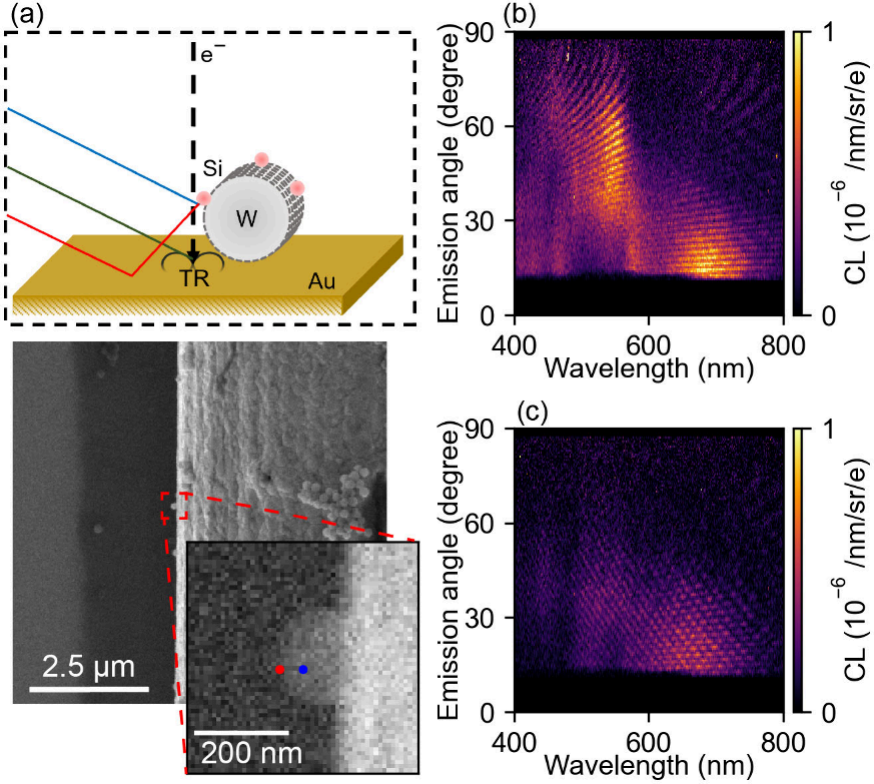
CL interferometry of a Si Mie particle. (a) Schematic representation and SEM image of the experimental configuration. An elevated Si Mie particle is excited by the e-beam. For off-particle excitation, the e-beam grazes the Si particle and subsequently excites the particle and TR at the Au surface. (b, c) Experimentally measured angle- and wavelength-resolved CL intensity for p-polarized CL emission for (b) on-particle excitation (corresponding to the blue dot in (a)) and (c) off-particle excitation (red dot in (a)).

Figure 5 shows the measured angle- and wavelength-resolved data for p-polarized CL emission for (b) on-particle and (c) off-particle excitation, corresponding to the blue and red dots in the inset of Figure 5a, respectively. For both configurations, we observe a clear angle dependence of the interference spectra. For on-particle excitation (Figure 5b), we see a similar interferogram as for the plasmonic tips: slow spectral oscillations at low emission angles and faster oscillations toward higher angles. However, in contrast to the earlier data, the intensity of the interferences is strongly modulated by the strong resonant nature of Mie scattering in the dielectric particles. We observe a strong magnetic dipole component around a wavelength of 700 nm that emits mostly toward low angles and a quadrupolar mode at 500 nm that emits over the entire angular range (except low angles). This matches well with the angular emission trends found in earlier work.^26,27^ For off-particle excitation, we observe an additional interference contribution due to the emission of TR at lower emission angles, as was also observed for the plasmonic nanotip. To reconstruct the height of this specific nanoparticle, we do the same analysis as we did for the example of the free-standing nanopillar, shown in Supporting Information S7. The resulting distance retrieved between the particle and the substrate is 9.18 μm.

In summary, we have demonstrated angle-resolved CL interferometry from resonant plasmonic scatterers in a geometry where a single electron coherently excites multiple plasmons. Free-standing plasmonic nanotips show interference with their mirror image, and in an excitation geometry where the electron is not intersected by the tip, TR from the substrate adds to the interference. The interferograms are in excellent correspondence with a coherent interference model that allows for the determination of the distance between the scatterers as well as the electron traveling time (and hence, the velocity). This method can be further expanded to a wider range of geometries. Polarization-resolved CL measurements further corroborate the validity of the interferometric scattering model. Replacing the nanotip by Si Mie scatterers, more complex interferograms are observed, modulated by the resonantly enhanced emission from the particles. The CL interferometry presented here opens new applications in the characterization of 3D nanostructures, in particular in the *z* direction, normal to the sample’s substrate, which is not easily accessible in an SEM.

The visibility of the observed interference fringes agrees well with the coherent summation of the radiation intensities from the individually excited sources. This implies that the dephasing of a tip plasmon in the time between its excitation and the generation of TR does not affect the interference, as expected from the nature of free-electron-driven excitations (i.e., they are independent of the electron’s temporal extension).^28^ Our experiments also inspire further studies to use the interference to separate coherent and incoherent contributions to CL emission, as the visibility of the interference fringes depends on the mutual coherence of the different sources involved. For example, the ratio between coherent and incoherent emission from a substrate (e.g., polaritonic vs inelastic emission) directly impacts the visibility of the interference fringes when the electron passes near a reference similar to the tip or Si sphere used in this work. Future work could extend the present analysis to semiconductor substrates, where incoherent CL becomes comparatively sizable (unlike the gold surfaces used in the present study).

## Supplementary Material



## Notes

An analytical model for the far-field interferogram, CL spectra of a free-standing nanopillar, and angle- and wavelength-resolved emission from TR next to a pillar; details of the fitting procedures for on-pillar and off-pillar excitation, distortions in the interferogram for large spacings, and Fourier analysis of Si particle interferometry; and experimental setup and methods, including angle- and wavelength-resolved CL measurements and fabrication of the plasmonic tip and dielectric sphere (PDF)
