# Supplementary material for: Angle-Resolved Cathodoluminescence Interferometry of Plasmonic and Dielectric Scatterers
Source: Nano Lett. 2025 Sep 18;25(39):14264–9. doi: 10.1021/acs.nanolett.5c02952 (PMC12492383; doi:10.1021/acs.nanolett.5c02952)
Supplement: Supplementary file 1 [file nl5c02952_si_001.pdf]

Angle-resolved cathodoluminescence interferometry of plasmonic and dielectric scatterers

Evelijn Akerboom[a*], Hiroshi Sugimoto[b], Minoru Fujii[b], F. Javier García de Abajo[c,d], Albert Polman[a*]

[a] Center for Nanophotonics, NWO-Institute AMOLF, Science Park 104, 1098 XG Amsterdam, the Netherlands

[b] Department of Electrical and Electronic Engineering, Graduate School of Engineering, Kobe University, Rokkodai, Nada, Kobe 657-8501, Japan

[c] ICFO-Institut de Ciencies Fotoniques, The Barcelona Institute of Science and Technology, 08860 Castelldefels, Barcelona, Spain

[d] ICREA-Institució Catalana de Recerca i Estudis Avançats, Passeig Lluís Companys 23, 08010 Barcelona, Spain

* Corresponding email: e.akerboom@amolf.nl, a.polman@amolf.nl

---

Contents

S1: Analytical model for far-field interferogram 2

S2: CL spectrum of a free-standing nanopillar 4

S3: Spectral and angular dependence of TR emission next to a nanopillar 5

S4: Fitting procedure for on-pillar excitation 6

S5: Fitting procedure for off-pillar excitation 8

S6: Distortions for large spacings in the interferogram 10

S7: Fourier analysis of the Si particle interferometry 12

S8: Experimental setup and methods 13

  Angle- and wavelength-resolved CL measurements 13

  Fabrication of the free-standing plasmonic tip 13

  Fabrication of the elevated dielectric sphere 13

References 14

S1: Analytical model for far-field interferograms

The total electric field ($\mathbf{E}_{\text{tot}}$) is given by the coherent sum of the field from three sources: transition radiation (TR) generated by the electron exciting the Au-coated substrate ($\mathbf{E}_{\text{TR}}$), direct plasmon radiation from the tip ($\mathbf{E}_{\text{tip}}$), and its reflection from the Au-coated substrate ($\mathbf{E}_{\text{tip,r}}$):

$$\mathbf{E}_{\text{tot}}(\mathbf{r},\omega) = \boldsymbol{E}_{\text{TR}}(\mathbf{r},\omega)e^{i\phi_0} + \mathbf{E}_{\text{tip}}(\,\mathbf{r}-\mathbf{r}',\omega) + \mathbf{E}_{\text{tip,r}}(\mathbf{r}+\mathbf{r}',\omega), \qquad \text{(S1.1)}$$

where

$$\phi_0 = \frac{2\pi c}{\lambda}\text{TOF} \qquad \text{(S1.2)}$$

is a phase term to take into account the time-of-flight (TOF) of the electron to travel from the tip to the surface (i.e., TOF= $\Delta z/v_e$, corresponding to a distance Δz and a velocity v_e). For the grazing excitation geometry, we model the radiation produced by the plasmonic nanotip as that of a Au nanoparticle with a radius of 75 nm, and both in-plane and out-of-plane electric dipole contributions are obtained using the analytical Mie theory-based model of electron-induced dipole excitation from Stamatopoulou *et al.*[1] for an electron impact parameter of 85 nm measured from the center of the particle. Light reflection at the Au surface is modeled using standard Fresnel reflection coefficients. The electric far field of the TR is calculated for an electron normally impinging a homogenous planar surface as[2]

$$\mathbf{E}_{\text{TR}}(\mathbf{r},\omega) = ik_0\cos(\theta)\,D\mu_1\frac{e^{ik_0 r}}{r}\,\hat{\boldsymbol{\theta}}, \qquad \text{(S1.3)}$$

where k_0 is the vacuum wavenumber ($k_0 = 2\pi/\lambda$) and D is given by

$$D = \frac{2ieq_{||}/c}{q_{z1}\epsilon_2 + q_{z2}\epsilon_1}, \qquad \text{(S1.4)}$$

with $\epsilon_{1,2}$ being the relative permittivity and q_{z1} and q_{z2} the perpendicular wave vector components in vacuum and Au, respectively. Here, $q_{||}$ is the in-plane wave vector of light in vacuum, and μ_1 given by

$$\mu_1 = \frac{1}{|v|}\left(\frac{\omega\epsilon_2 + vq_{z2}\epsilon_1}{q^2 - k^2\epsilon_1} - \frac{\omega\epsilon_1 - vq_{z1}\epsilon_2}{q^2 - k^2\epsilon_2}\right). \qquad \text{(S1.5)}$$

The angle- and frequency-dependent CL emission intensity (in units of number of photons per electron per photon-energy bandwidth per srad) is then given by

$$\text{CL}_{\text{tot}}(\theta,\mathbf{r}) = \frac{c}{4\pi^2\hbar\omega}|\mathbf{E}_{\text{tot}}|^2\,r^2\sin(\theta) \qquad \text{(S1.6)}$$

The calculated CL interferogram and its Fourier transform (from frequency to time) for each emission angle are shown in Figure 2e,f.

We approximate our plasmonic nanotip as a Au nanosphere with a radius of 75 nm that only supports an electric dipole at the wavelength range under consideration. For the on-tip excitation, we use an impact parameter of 10 nm, relative to the particle center. For the off-tip excitation, an impact parameter of 85 nm is used. We incorporate optical constants from Olman et al.[3] for Au.

To model the electron-induced radiation spectra from plasmonic and dielectric Mie particles, we use an analytical model from Stamatopoulou *et al.*[1] (also developed independently by Matsukata *et al.*[4]). The electric field at a frequency $\omega = 2\pi c/\lambda$ and position $\mathbf{r} = (r, \theta, \phi)$ is given by

$$\mathbf{E}_{\text{tip}}(\mathbf{r}, \omega) \approx \sum_{m=-1}^{1} \frac{i}{k_0} a_{1m} \nabla \times h_1^{(+)}(k_0 r) \mathbf{X}_{1m}(\theta, \phi) \tag{S1.7}$$

for the contribution of components with dipolar, transverse-electric (TE) symmetry. Here, a_{1m} are the corresponding expansion coefficients, which depend on particle radius, electron velocity (v_e), and impact parameter (b). Furthermore, $h_1^{(+)}(s) = (1/s - i)\, e^{is}/s$ is a spherical Hankel function of the first kind, k_0 is the free space wavenumber ($k_0 = 2\pi/\lambda$), and $\mathbf{X}_{1m}(\theta, \phi)$ are the spherical harmonics defined as

$$\mathbf{X}_{1m}(\theta, \phi) = \frac{1}{\sqrt{2}} \mathbf{L} Y_{1m}(\theta, \phi), \tag{S1.8}$$

where $Y_{1m}(\theta, \phi)$ are spherical harmonics and $\mathbf{L} = -i\mathbf{r} \times \boldsymbol{\nabla}$ is the angular momentum operator.

S2: CL spectrum of a free-standing nanopillar

Figure S2 shows CL spectra for the free-standing tip of a nanopillar. In particular, Figure S2a shows the CL map at a wavelength of 600 nm and a bandwidth of 20 nm, clearly revealing an enhanced emission at the tip. Figure S2b shows the CL spectrum at two impact parameters: at the tip (blue) and at the shaft (purple).

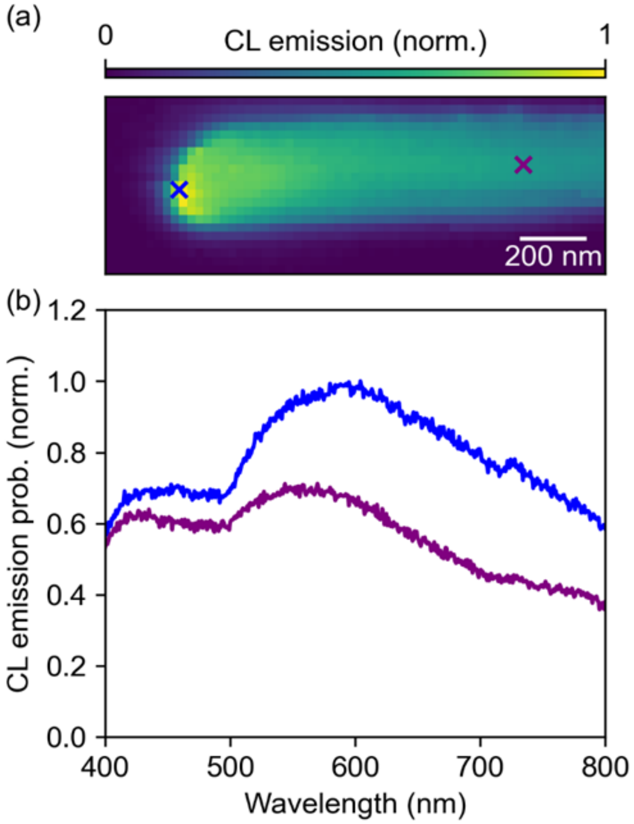

Figure S 2 CL spectrum of a free-standing nanopillar. (a) CL emission map of the nanopillar at a wavelength of 600 nm with a bandwidth of 20 nm. (b) CL spectra for two impact parameters: at the tip (blue) and at the shaft (purple), corresponding to the crosses in (a).

S3: Spectral and angular dependence of TR emission next to a nanopillar

Figure S3 shows the normalized wavelength and angle distribution of TR emission from a gold surface for an electron passing 100 nm away from a nanopillar, indicated by the red dot in Figure S3a. In Figure S3b, we find the absence of interference, indicating that light scattering from the shaft is not significant.

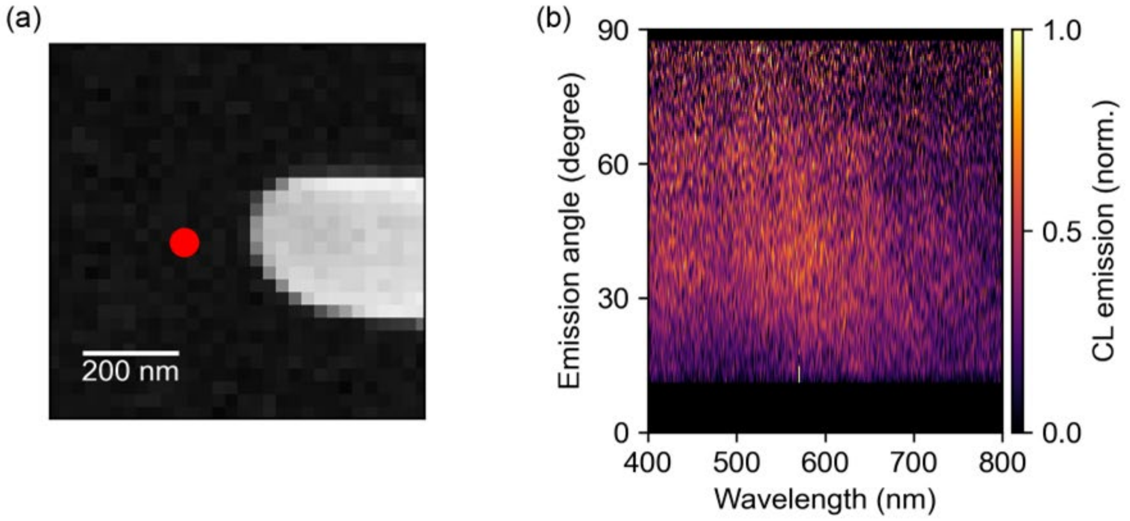

Figure S 3 Spectral and angular dependence of TR emission excited 100 nm away from the nanopillar. (a) Top-view SEM image of the nanopillar, with the red dot indicating the excitation spot of the electron. (b) Corresponding spectral and angular dependence of CL emission.

S4: Fitting procedure for on-pillar excitation

To reconstruct the characteristic dimensions of our geometry, we analyze the interferogram normalized to the spectral response corresponding to Figure S4a. We take the discrete inverse Fourier transform according to

$$\mathrm{x}_k = \frac{1}{N}\sum_{n=0}^{N} X_n e^{\frac{2i\pi kn}{N}}. \tag{S4.1}$$

The Fourier transform in Figure S4b shows clear branches corresponding to the emission of the tip interfering with its reflection at the substrate. The angle-dependent time delay of the branch follows the relation

$$\Delta t(\theta) = \frac{2\Delta z\sin(\theta)}{\mathrm{c}}. \tag{S4.2}$$

To recover the height (Δz) from the Fourier transform, we fit the image to three Lorentzian peaks: one at t=0 and the other two at $t = \pm\Delta t$, as given by

$$y(t,\theta) = \frac{A_0(\theta)\gamma_0^2}{\gamma_0^2 + t^2} + \frac{A_1(\theta)\gamma_1^2}{\gamma_1^2 + (t-\Delta t)^2} + \frac{A_1(\theta)\gamma_1^2}{\gamma_1^2 + (t+\Delta t)^2}, \tag{S4.3}$$

with γ_0 and γ_1 the temporal widths of the Lorentzians, treated as global parameters, and $A_0(\theta)$ and $\mathrm{A}_1(\theta)$ the angle-dependent amplitude of the interference. The resulting fit is shown in Figure S4d, producing $\Delta z = 5.370\ \mu m$, $\gamma_0 = 0.7\ fs$, and $\gamma_1 = 1.94\ fs$. The residuals (data minus fitted data) are shown in Figure S4c and we see that the fit is rather accurate, with some characteristic features observed. First, the horizontal red lines correspond to artifacts that occur when the Fourier transform of a finite spectral domain is taken. Furthermore, around the branches, the residuals are a few percent, either positive or negative. This is due to the limited temporal resolution of 2.3 fs. If we examine line traces at specific angles in Figure S4e, corresponding to emission angles of 20°, 50°, and 80° in blue, orange, and green, respectively, we observe that the fit nicely captures the data. Finally, from the fitted amplitudes, we can extract the angle-dependent visibility ($\nu(\theta)$) of the fringes as

$$\nu(\theta) = \frac{A_1(\theta)}{A_0(\theta)}, \tag{S4.4}$$

shown in Figure S4f, and we find a visibility of around 15%.

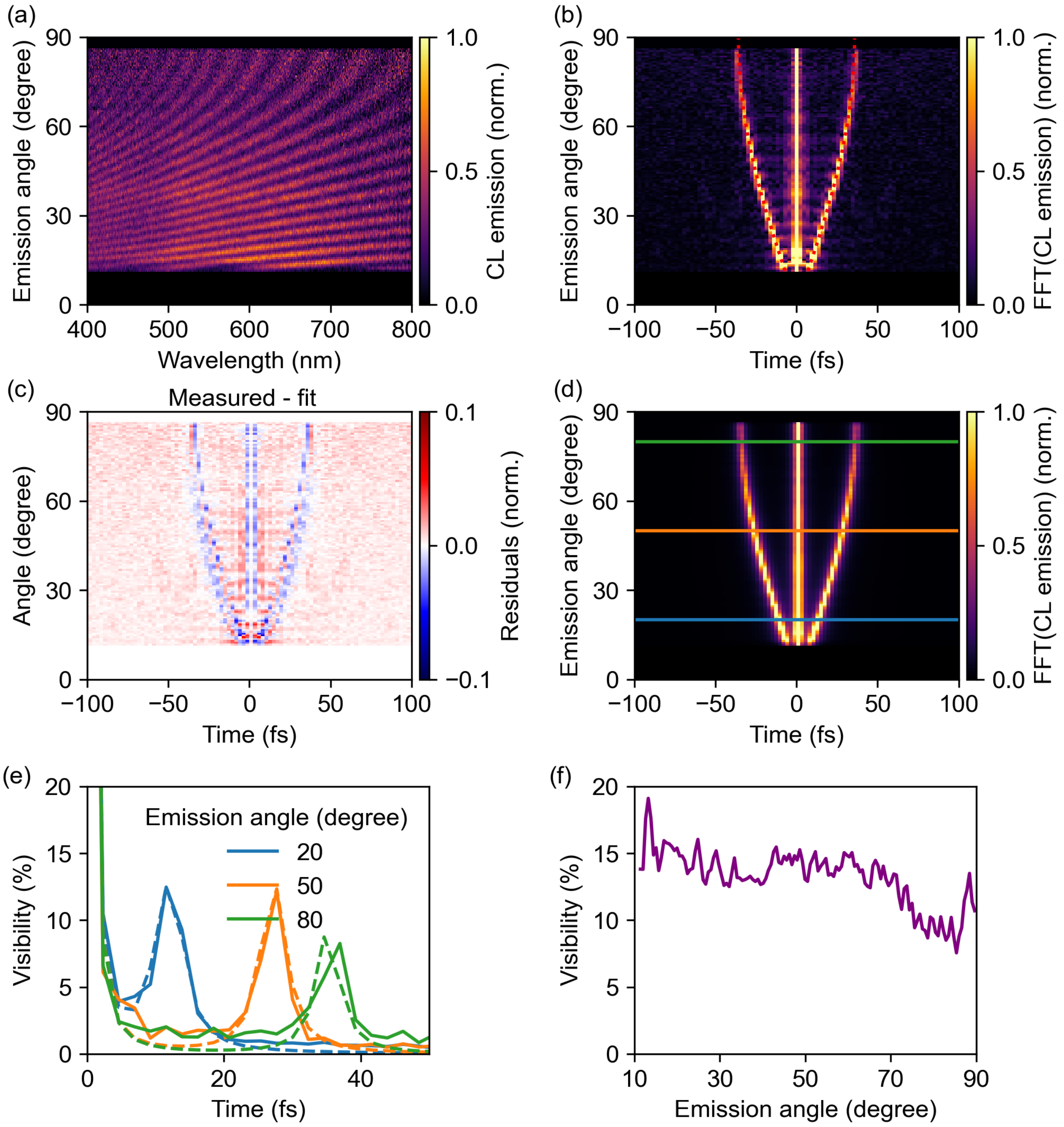

Figure S 4 Fitting procedure for the on-tip excitation. (a) Spectrally normalized interferogram and (b) its Fourier transform, (d) the fit of (b) to three Lorentzians as given by eq S4.3, and (c) the residuals of the data with the fit. Panel (e) shows line traces of the experimental data (b) and the fit (d) as solid and dashed curves, respectively, for three specific emission angles: 20° (blue), 50° (orange), and 80° (green). Panel (f) shows the angle-dependent visibility of the interference given by eq S4.4.

S5: Fitting procedure for off-pillar excitation

To reconstruct the characteristic dimensions of our geometry, we analyze the interferogram normalized to the spectral response corresponding to Figure S5a. The Fourier transform in Figure S5b shows clear branches corresponding to the emission of the tip interfering with its reflection at the substrate (inner one), the interference between the reflection of the tip and TR (second one), and between the tip and TR (outer one). The angle-dependent time delay of the outer branches follows the relation

$$\Delta t_{\pm}(\theta) = \frac{\Delta z}{v_e} \pm \frac{\Delta z \sin(\theta)}{c}, \qquad \text{(S5.1)}$$

with v_e the speed of the electron. To recover the height (Δz) from the Fourier transform, we fit the image to seven Lorentzian peaks: one at t=0 and the other ones at $t = \pm\Delta t$:

$$\begin{aligned} y(t,\theta) = {} & \frac{A_0(\theta)\gamma_0^2}{\gamma_0^2 + t^2} + \frac{A_1(\theta)\gamma_1^2}{\gamma_1^2 + (t-\Delta t)^2} + \frac{A_1(\theta)\gamma_1^2}{\gamma_1^2 + (t+\Delta t)^2} \\ & + \frac{A_2(\theta)\gamma_2^2}{\gamma_2^2 + (t+\Delta t_+)^2} + \frac{A_2(\theta)\gamma_2^2}{\gamma_2^2 + (t-\Delta t_+)^2} \\ & + \frac{A_3(\theta)\gamma_3^2}{\gamma_3^2 + (t-\Delta t_-)^2} + \frac{A_3(\theta)\gamma_3^2}{\gamma_3^2 + (t+\Delta t_-)^2}, \end{aligned} \qquad \text{(S5.2)}$$

with γ_0, γ_1, γ_2, and γ_3 the temporal widths of the Lorentzians, and $A_0(\theta), A_1(\theta), A_2(\theta)$, and $A_3(\theta)$ the angle-dependent amplitude of the interfering terms. The resulting fit is shown in Figure S5d, and we retrieve a slightly smaller height than before: $\Delta z = 5.340\ \mu m$. The other fitting parameters are shown in table S5.1.

Table 5.1. Resulting fitting parameters of the fit shown in Figure 5d, along with the width and visibility of the different interference components resulting in the interferogram of Figure S5a.

Fitting parameter	*center*	*Tip with reflection*	*Reflection with TR*	*Tip with TR*
Width (γ_i)	0.70 fs	1.95 fs	1.48 fs	1.65 fs
Visibility (A_i/A_0)	n/a	10-15 %	20-10%	20-5%

The residuals (data minus fitted data) are shown in Figure S5c. Together with the line traces at specific angles in Figure S5e, corresponding to emission angles of 20°, 50°, and 80° in blue, orange, and green, respectively, we find that the fit nicely captures the data. Finally, we can compare the visibility of the different components in Figures S5f: the tip with its reflection (purple), the reflection with TR (red), and the tip with TR (pink). We note here that, at high emission angle, from 70° onwards, the contribution from the tip with its reflection and the reflection with TR are merging into a single peak, making them indistinguishable. However, in the branch corresponding to the tip interfering with TR (pink), we observe that the visibility clearly decreases from 20% to 0% for emission angles from 60° to 90°, corresponding to the characteristic angular emission of TR.

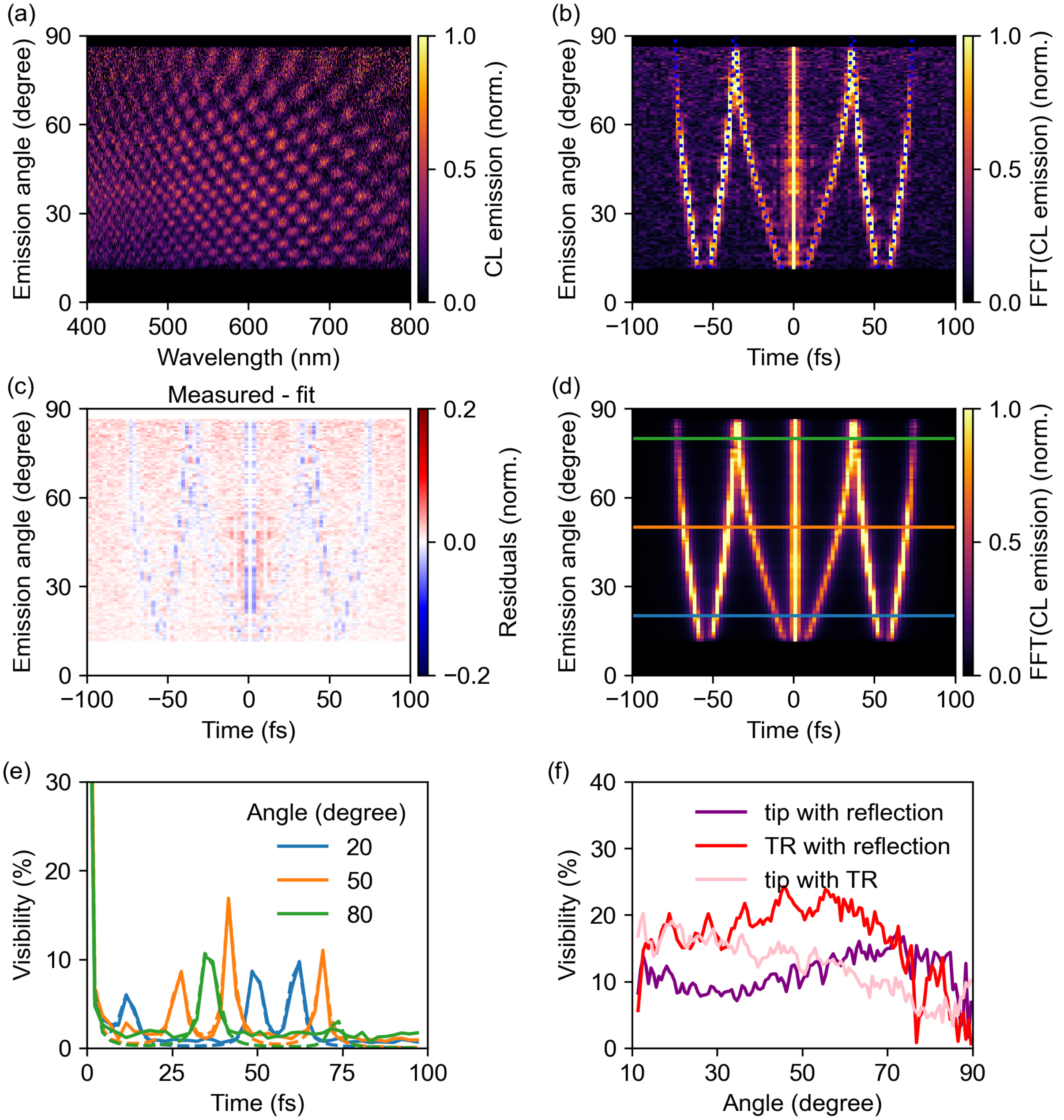

Figure S 5 Fitting procedure for the off-tip excitation. (a) Spectrally normalized interferogram and (b) its Fourier transform, (d) the fit of (b) to seven Lorentzians as given by eq S5.2, and (c) the residuals of the data with the fit. Panel (e) shows line traces of the data (b) and the fit (d) as solid and dashed curves, respectively, for three specific emission angles: 20° (blue), 50° (orange), and 80° (green). Panel (f) shows the angle-dependent visibility of the interference for the center peak (purple), the first side peak (red), and the second side peak (pink).

S6: Distortions in the interferogram for large spacings

Since we are working with relatively large spacings in this work, of the order of a few microns, we have to consider the effect on the light collection by the parabolic mirror. In our calculations, we assume small angles: the mirror is positioned so far away from the sample relative to the height of the pillar (R >> Δz) that we treat the light rays from the tip and from its reflection as parallel rays and use eq. S4.2 to derive the time difference at the mirror. However, in practice, the distance between the sample surface and the mirror is of a few millimeters. While this is still large compared to the optical wavelength, for large pillar height, the parallel ray approximation breaks down. This has two main consequences on the measured interferogram. First of all, this slightly modifies the time difference between the sources (e.g., for a 5 μm high tip, this effect amounts to 2e-5 fs at its maximum). Secondly, there is a modification to the emission angle. Although this angular deviation is small (only a few mrad), it propagates through the collection system and leads to a significant error for large spacing. Figure S6a shows a schematic representation of this effect, for an exaggerated tip spacing of 6 mm: the ray from the surface (black dashed) reflects horizontally, while the ray from the tip (red) is displaced lower on the detector, and the ray from the reflection (green) is displaced higher on the detector.

Next, we calculate this difference and estimate the expected error. We use the expression of the parabolic mirror at the azimuthal angle ($\phi = 0$), given by

$$x(z) = az^2 - \frac{1}{4a} \,, \tag{S6.1}$$

with $a = 0.1 \text{ mm}^{-1}$. A real-space schematic is shown in Figure S6a, indicating the emission angle (θ_0), which is given by

$$\theta_0(z) = \tan\left(\frac{z}{x}\right). \tag{S6.2}$$

The angles for the tip emission (θ_+) and the reflection emission (θ_-) are given by

$$\theta_\pm(z) = \tan\left(\frac{z \pm \Delta z}{x}\right). \tag{S6.3}$$

Since the incoming angle equals the outgoing angle for a mirror, the deviation on the reflected angle is given by the difference in emission angle,

$$\alpha_\pm = \theta_\pm(z) - \theta_0(z). \tag{S6.4}$$

As a result, the displacement on the detector reads

$$d_z = \frac{d_x \tan(\alpha_\pm)}{A}, \tag{S6.5}$$

with d_x the propagation length (40 cm), and A the magnification ($A = 3$). The resulting distortion on the emission angle measured on the detector is shown in Figure S6b for pillar heights up to 10 μm. We can clearly observe that this distortion is worse for low emission angles, while for higher emission angles the result is still correct even for pillars of 10 μm in height. Next, in Figure S6c,d, we show the resulting interferogram and corresponding Fourier transform for a spacing of 10 μm, along with the expected temporal delay, overlayed in white dashed.

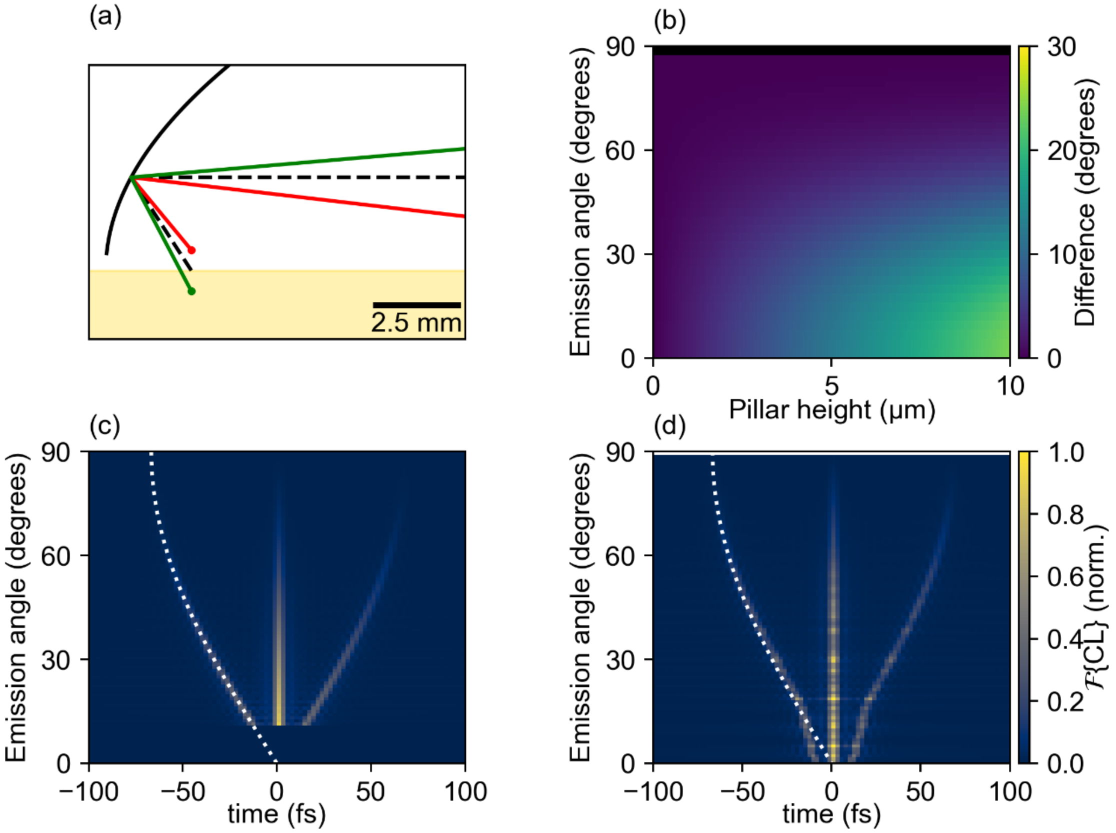

Figure S 6 Distortion of the interference due to the limited focal area of the parabolic mirror. (a) Exaggerated schematic of the rays from different points reflecting of the mirror, for two points sources at ± 0.5 mm from the focus. (b) Calculated error in the measured angle as a function of scatterer displacement and emission angle. (c,d) Temporal dependence for two scatterers that are vertically spaced by 20 μm, calculated according to the model of eq.S1.1 (c) without and (d) with the correction on the angular distortion. The white-dashed lines denote the uncorrected time delay expected according to eq S4.2.

S7: Fourier analysis of the Si particle interferometry

Figure S7 shows the interferogram for a Si particle above a gold substrate excited by an electron beam (e-beam). To reconstruct the height of this specific nanoparticle, we take the Fourier transform of the angle- and wavelength-resolved spectra. This is shown in Figure S7(c,e) for on-particle and off-particle configurations, respectively. In the Fourier-transformed data, we observe the same behavior as we found in the examples using a gold nanopillar: if the electron impacts the particle in the center, only the central branches are obtained; in contrast, for off-particle excitation, outer branches are present, indicating that TR is excited and interferes with the emission from the particle. Yet, there are notable differences compared to the plasmonic nanotip.

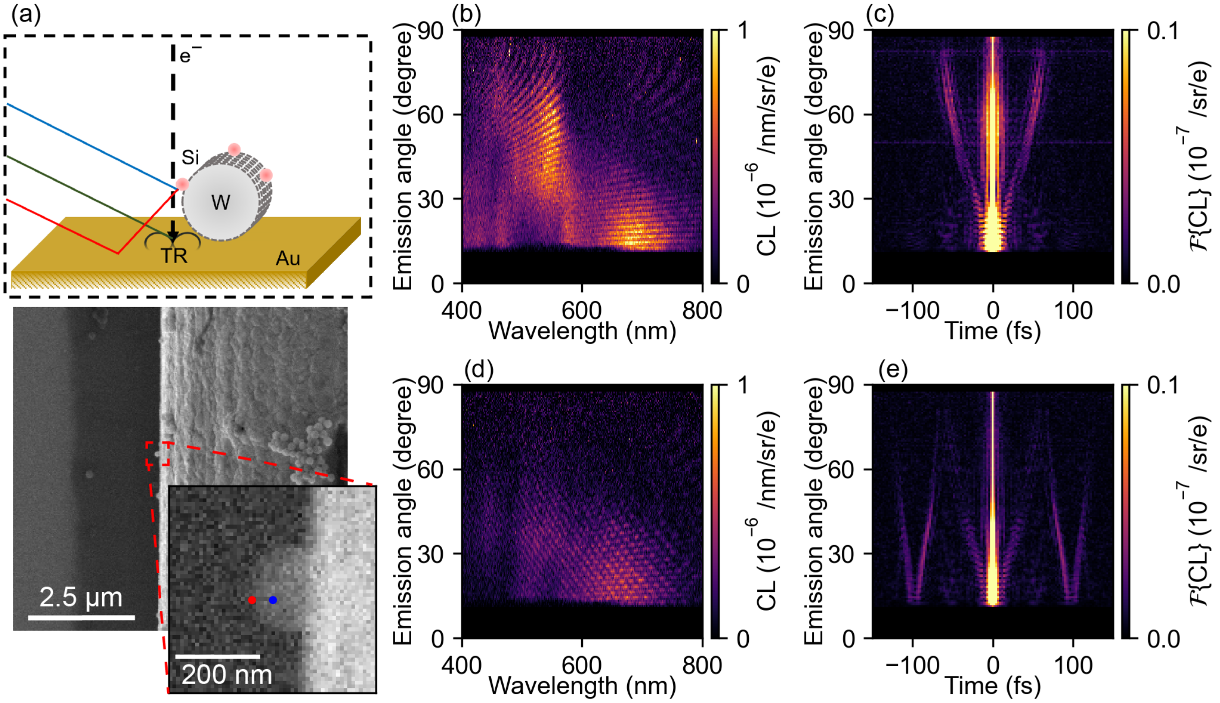

Figure S7 CL interferometry of a Si Mie particle (same data as Figure 5). (a) Schematic representation and SEM image of the experimental configuration. An elevated Si Mie particle is excited by the e-beam. For off-particle excitation, the e-beam grazes the Si particle and subsequently excites TR at the Au surface. (b,d) Experimentally measured angle- and wavelength-resolved CL intensity for p-polarized CL emission for (b) on-particle excitation (corresponding to the blue dot in (a)) and (d) off-particle excitation (red dot in (a)). (c,e) Fourier transform of (b) and (d) to the time domain.

First of all, the central branch deviates from the expected sinusoidal behavior, particularly at low emission angles. Instead, it shows a plateau with a nearly constant time delay up to approximately 20°, beyond which the delay begins to increase. This is a result of the parabolic shape of the mirror: at low emission angles, there is a large effect of the particle being out of focus, especially for these high systems; as the particle (and thus also its reflection) is not in the focal position of the mirror anymore, light does not come out in parallel directions, and this creates distortions in the interferogram. This behavior aligns well with numerical predictions (see Supporting Information Figure S6). Therefore, we use the time delay at high emission angles to reconstruct the height of this particle. At 85°, the time delay of the center branch is 61 fs, which corresponds to a particle height of 9.18 μm. Secondly, the temporal width of the branches is

significantly broader: several femtoseconds rather than the ~1 fs width that was previously observed. This broadening reflects the resonant response of the silicon nanoparticle, which supports various Mie modes (MD, ED, MQ, EQ). Each resonance has a distinct quality factor, leading to different lifetimes and, thus, temporal broadening in the Fourier domain.

S8: Experimental setup and methods

Angle- and wavelength-resolved CL measurements

Cathodoluminescence (CL) measurements were conducted in an FEI Quanta FEG 650 scanning electron microscope (SEM; Thermo Fisher Scientific Inc., MA, USA) equipped with a Schottky electron source. To collect the CL emission, the setup includes a parabolic mirror positioned between the sample and the electron column, directing the light into an optical detection system (SPARC Spectral, DELMIC BV, The Netherlands)[5]. Angle- and wavelength-resolved CL measurements were performed by positioning a vertical slit of 100 µm width in the image plane to only select CL from a narrow azimuthal angular emission range. The CL is dispersed by a diffraction grating to retrieve spectral information. To measure the polarization-dependent CL emission, a horizontal or vertical polarizer is used to filter p- and s-polarized light, respectively.[6] The measurements were performed with an e-beam current of 1.4 nA and either 30 keV or 20 keV acceleration voltage.

For the measurements on the plasmonic tip, an acquisition time of 360 s was chosen for the full angular and spectral interferogram. A line scan with pixels of 10 nm was made to control the position of the e-beam close to the tip. For the dielectric particles, a 2D map was made with pixels of 50 nm and 240 s acquisition time. For all measurements, a dark measurement with the same settings was subtracted and the data was corrected for the system's response. The latter was calibrated using TR emission from a single-crystal aluminum sample as a reference.[7]

Fabrication of the free-standing plasmonic tip

As a substrate, a single-crystalline polished Si (100) substrate was used. First, the substrate was cleaned using base-piranha cleaning and sputter-coated with 2 nm chrome adhesion layer and a 50 nm thick Au film (EM ACE600, Leica, Inc.). Next, a free-standing Pt nanopillar was grown using e-beam-induced deposition (EBID) in the SEM (FEI Helios NanoLab 600, Thermo-Fisher Scientific, Inc.). EBID was done with an e-beam of 2-keV energy and a current of 170 pA at 45° tilt angle and a dwell time of 1.5 ms. The base of the pillar was 300 nm, decreasing to a diameter of 40 nm at the tip. After the growth, the entire sample was covered with a 50 nm thick layer of Au through sputter-coating. This resulted in a free-standing Au nanotip above a 100-nm thick Au film.

Fabrication of the elevated dielectric sphere

Silicon nanospheres were fabricated by crushing SiO lumps to a powder. The powder was annealed in N_2 and etched with hydrofluoric acid (HF). The single Si nanospheres were suspended in methanol, ultrasonicated, and filtered to a particle size of around 200 nm diameter.[8] As a substrate, the same fabrication was used as for the free-standing plasmonic tip. The elevated dielectric spheres were fabricated using a 10-µm-diameter W wire. First, 1 cm of W wire was cleaned with isopropanol (IPA) and dried using N_2 gas. Next, a wire was deposited in a droplet of a suspension of Si nanoparticles in IPA on a glass substrate. When all IPA was evaporated, the wire was taken from the substrate and rinsed in IPA. Next, the wire was

deposited on the Au-coated Si substrate with a 10 µL droplet of water to ensure that the wire was attached to the surface. This was dried at 60°C, resulting in elevated Si nanospheres around 5 µm above the Au surface, depending on the location of the nanosphere with respect to the curvature of the wire.

References

(1) Stamatopoulou, P. E.; Zhao, W.; Rodríguez Echarri, Á.; Mortensen, N. A.; Busch, K.; Tserkezis, C.; Wolff, C. Electron Beams Traversing Spherical Nanoparticles: Analytic and Numerical Treatment. *Phys Rev Res* **2024**, *6* (1), 1–15. https://doi.org/10.1103/PhysRevResearch.6.013239.

(2) García de Abajo, F. J. Optical Excitations in Electron Microscopy. *Rev Mod Phys* **2010**, *82* (1), 209–275. https://doi.org/10.1103/RevModPhys.82.209.

(3) Olmon, R. L.; Slovick, B.; Johnson, T. W.; Shelton, D.; Oh, S.-H.; Boreman, G. D.; Raschke, M. B. Optical Dielectric Function of Gold. *Phys Rev B* **2012**, *86* (23), 235147. https://doi.org/10.1103/PhysRevB.86.235147.

(4) Matsukata, T.; Matthaiakakis, N.; Yano, T. A.; Hada, M.; Tanaka, T.; Yamamoto, N.; Sannomiya, T. Selection and Visualization of Degenerate Magnetic and Electric Multipoles up to Radial Higher Orders by Cathodoluminescence. *ACS Photonics* **2019**, *6* (9), 2320–2326. https://doi.org/10.1021/acsphotonics.9b00833.

(5) Coenen, T.; den Hoedt, S. V.; Polman, A. A New Cathodoluminescence System for Nanoscale Optics, Materials Science, and Geology. *Micros Today* **2016**, *24* (3), 12–19. https://doi.org/10.1017/s1551929516000377.

(6) Coenen, T.; Polman, A. Cathodoluminescence Fourier Microscopy. *Opt Express* **2012**, *20* (17), 18679–18691.

(7) Coenen, T.; Vesseur, E. J. R.; Polman, A. Angle-Resolved Cathodoluminescence Spectroscopy. *Appl Phys Lett* **2011**, *99* (14), 2009–2012. https://doi.org/10.1063/1.3644985.

(8) Sugimoto, H.; Okazaki, T.; Fujii, M. Mie Resonator Color Inks of Monodispersed and Perfectly Spherical Crystalline Silicon Nanoparticles. *Adv Opt Mater* **2020**, *8* (12), 2000033. https://doi.org/10.1002/adom.202000033.
